# New-Onset Tic Disorder Associated With Bupropion XL: A Rare Case

**DOI:** 10.7759/cureus.102855

**Published:** 2026-02-02

**Authors:** Ovais Rashid, Priyanka Hooda, Simranjeet Kaur

**Affiliations:** 1 Centre of Excellence in Mental Health, Psychiatry, Atal Bihari Vajpayee Institute of Medical Sciences (ABVIMS) and Dr. RML Hospital, New Delhi, IND; 2 Clinical Psychiatry, University of South Wales, Cardiff, GBR; 3 Cardiology/Critical Care, Sant Parmanand Hospital Civil Lines, New Delhi, IND

**Keywords:** adverse drug reaction, bupropion, depression, norepinephrine-dopamine reuptake inhibitor (ndri), tic

## Abstract

Tics are sudden, rapid, non-rhythmic movements or vocalizations. Bupropion XL, a norepinephrine-dopamine reuptake inhibitor (NDRI), is approved for depression and smoking cessation. While tics are a known adverse effect of stimulants and have been rarely reported with selective serotonin reuptake inhibitors (SSRIs), they are exceptionally uncommon with bupropion. We present the case of a 27-year-old male patient with no prior history of tic disorder who developed motor (clapping) and vocal (whistling, laughing) tics following the initiation of bupropion XL for depressive symptoms. The tics subsided completely upon discontinuation of bupropion and initiation of low-dose risperidone. This case highlights a rare but clinically significant adverse effect of bupropion, underscoring the need for clinical vigilance when prescribing this agent.

## Introduction

Tics are sudden, rapid, recurrent, non-rhythmic motor movements or vocalizations, typically preceded by a premonitory urge [1]. Their pathophysiology involves dysfunction of the cortico-striato-thalamo-cortical (CSTC) circuits, with dopaminergic hyperactivity playing a central role [2]. Several pharmacological agents, particularly those that affect dopaminergic transmission, can induce or exacerbate tics. These include psychostimulants (e.g., methylphenidate), certain anticonvulsants, and, more rarely, antidepressants [3].

Among antidepressants, case reports have documented tic induction with selective serotonin reuptake inhibitors (SSRIs), including fluoxetine, sertraline, and escitalopram [4]. Bupropion, an atypical antidepressant that acts as a norepinephrine-dopamine reuptake inhibitor (NDRI), is generally considered to have a favorable movement disorder profile. However, its dopaminergic activity poses a theoretical risk. To date, only one prior case report has described new-onset tics associated with bupropion XL (extended-release formulation) [5], with another reporting exacerbation of pre-existing tics [6]. We report a second case of new-onset mixed motor and vocal tics temporally associated with bupropion XL therapy, adding to the limited evidence supporting a possible adverse drug reaction.

## Case presentation

A 27-year-old male patient presented to the outpatient psychiatry clinic with a six-month history of involuntary clapping, whistling, and episodic uncontrollable laughter. These symptoms caused him significant social distress. His history revealed that he had been prescribed bupropion XL (titrated to 300 mg/day) and clonazepam (0.25 mg/day) at another facility eight months prior for symptoms of depression, including sad mood, anhedonia, insomnia, and poor appetite. The involuntary movements and sounds began approximately two months after the initiation of bupropion XL therapy. At presentation, he also reported residual depressive symptoms: low mood, anhedonia, fatigue, poor concentration, and low self-esteem. There was no past personal or family history of tic disorders, obsessive-compulsive disorder, or other major psychiatric illness. His medical history was significant for well-controlled HIV infection, maintained on a fixed-dose combination of tenofovir alafenamide, emtricitabine, and dolutegravir for four years with excellent adherence, and an incidental, stable arachnoid cyst with non-specific white matter changes (leukoencephalopathy) of indeterminate etiology, which had remained clinically silent and radiologically stable for years on prior neuroimaging.

A comprehensive physical and neurological examination was unremarkable, except for the observed motor and vocal tics. The motor tic presented as intermittent, abrupt, and repetitive bilateral clapping. The vocal tics consisted of simple, stereotyped whistling and involuntary laughter. These phenomena occurred multiple times per hour, were brief in duration, and could be suppressed with conscious effort for short periods. They were exacerbated by anxiety and social stress but did not disappear with distraction. No complex motor or vocal tics (e.g., copropraxia, echolalia) were observed.

Diagnostic investigations were undertaken to rule out alternative causes. Routine laboratory tests, including complete blood count, renal and liver function tests, and electrolytes, were all within normal limits (Table 1). A review of prior neuroimaging (brain MRI) confirmed the presence of a chronic, stable arachnoid cyst of indeterminate etiology and non-progressive white matter changes, which had remained clinically silent and radiologically stable for years and were deemed incidental and unrelated to the acute presentation. The case was discussed with a consulting neurologist. Given the clear temporal correlation with bupropion initiation, the absence of focal neurological deficits, normal routine laboratory investigations, and stable chronic findings on neuroimaging, further investigations such as cerebrospinal fluid analysis or electromyography/nerve conduction studies were not deemed clinically necessary. The patient's HIV was well-controlled, with an undetectable viral load and a CD4 count of 580 cells/μL.

**Table 1:** Detailed laboratory investigations. Normal reference ranges are based on standardized adult male values from institutional clinical laboratory standards.

Parameter (unit)	Patient's value	Normal reference range	Clinical note/interpretation
Complete blood count			
Hemoglobin (g/dL)	13.8	13.0-17.0	Within normal limits (WNL)
Hematocrit (%)	41	40-52	WNL
Total leukocyte count (cells/µL)	6,500	4,000-11,000	WNL
Neutrophils (%)	62	40-75	WNL
Lymphocytes (%)	28	20-50	WNL
Platelet count (x10³/µL)	220	150-450	WNL
Renal function test (KFT)			
Serum creatinine (mg/dL)	0.9	0.7-1.3	WNL
Blood urea nitrogen (mg/dL)	14	7-20	WNL
Estimated GFR (mL/min/1.73 m²)	>90	>60	WNL
Liver function test (LFT)			
Alanine aminotransferase (ALT) (U/L)	30	7-55	WNL
Aspartate aminotransferase (AST) (U/L)	28	8-48	WNL
Alkaline phosphatase (ALP) (U/L)	85	40-129	WNL
Total bilirubin (mg/dL)	0.8	0.2-1.2	WNL
HIV disease monitoring			
CD4 count (cells/µL)	580	500-1,200	WNL; indicates stable immune status
HIV-1 viral load (copies/mL)	Undetectable	Target: undetectable	Confirms effective viral suppression on highly active antiretroviral therapy (HAART)

The tics were present for most of the day and worsened during periods of stress. A diagnosis of drug-induced tic disorder secondary to bupropion XL was considered. Causality was assessed using the Naranjo Adverse Drug Reaction Probability Scale [7], a standard tool freely available for academic and clinical use; the score was 7, indicating a "probable" relationship. Bupropion XL was tapered and discontinued. Given the persistence and distress caused by the tics, risperidone 2 mg once daily was initiated. For his ongoing depressive symptoms, a low-dose SSRI (escitalopram 10 mg/day) was started.

Within three weeks of bupropion discontinuation, a noticeable reduction in the frequency and intensity of both motor and vocal tics was observed. After six weeks, the tics had reduced by over 70%. At the two-month follow-up, the tics had resolved completely. The patient's depressive symptoms also showed significant improvement on the Hamilton Depression Rating Scale (HAM-D), a clinician-administered tool in the public domain and freely available for clinical and academic use [8]. Risperidone was maintained at 2 mg, and the patient continues to be monitored regularly.

## Discussion

This case illustrates the induction of a mixed motor and vocal tic disorder in an adult patient following treatment with bupropion XL, supporting a rare but probable causal link based on temporal association and Naranjo scoring. The temporal relationship (onset after drug initiation, resolution after discontinuation), absence of personal or family history, and exclusion of other causes strongly support bupropion as the culprit. The neurobiological mechanism likely involves bupropion's inhibition of dopamine reuptake. Increased synaptic dopamine in the striatum can potentiate the activity of the CSTC pathways, predisposing individuals to develop tics [2,9]. Animal studies have shown that bupropion can induce stereotypical behaviors, supporting its potential to cause repetitive movements [10]. While SSRIs are more serotonergic, their rare association with tics may involve complex serotonergic modulation of dopaminergic tone [4]. Our case aligns with a single previous report that described identical symptoms (motor and vocal tics) in a patient without prior history after starting bupropion XL [5]. Another report described an exacerbation of a pre-existing tic disorder with bupropion [6]. Our patient’s concurrent medical conditions (HIV, arachnoid cyst) were stable and long-standing, making them unlikely primary causes for the acute onset of tics, though they may have contributed to an underlying neurological vulnerability. This adverse effect underscores the importance of considering a patient's neurological history when prescribing dopaminergic agents. Clinicians should inquire about personal or family history of tics or related disorders (e.g., OCD) before initiating bupropion and monitor for the emergence of abnormal movements during treatment.

## Conclusions

This case adds to the very limited literature suggesting a temporal association between bupropion XL therapy and the emergence of new-onset, mixed motor and vocal tics. The close temporal relationship (onset after initiation and resolution after discontinuation) and a high Naranjo score indicate a probable adverse drug reaction, mechanistically plausible given bupropion's dopaminergic activity. Clinicians should be aware of this potential association, consider screening for personal or family history of tic disorders before prescription, and monitor for abnormal movements. The emergence of new tics in a patient on bupropion should prompt consideration of a drug-induced etiology, as discontinuation typically leads to resolution. Further pharmacovigilance and research are needed to better understand this rare potential risk.
