# Correction: New-Onset Tic Disorder Associated With Bupropion XL: A Rare Case

**DOI:** 10.7759/cureus.c406

**Published:** 2026-02-20

**Authors:** Ovais Rashid, Priyanka Hooda, Simranjeet Kaur

**Affiliations:** 1 Centre of Excellence in Mental Health, Psychiatry, Atal Bihari Vajpayee Institute of Medical Sciences (ABVIMS) and Dr. RML Hospital, New Delhi, IND; 2 Clinical Psychiatry, University of South Wales, Cardiff, GBR; 3 Cardiology/Critical Care, Sant Parmanand Hospital Civil Lines, New Delhi, IND

This article has been corrected to fix three typos introduced by the editor in the Normal Reference Range column of Table 1.

